# Antineoplastic Drug-Induced Cardiotoxicity: A Redox Perspective

**DOI:** 10.3389/fphys.2018.00167

**Published:** 2018-03-07

**Authors:** Gilda Varricchi, Pietro Ameri, Christian Cadeddu, Alessandra Ghigo, Rosalinda Madonna, Giancarlo Marone, Valentina Mercurio, Ines Monte, Giuseppina Novo, Paolo Parrella, Flora Pirozzi, Antonio Pecoraro, Paolo Spallarossa, Concetta Zito, Giuseppe Mercuro, Pasquale Pagliaro, Carlo G. Tocchetti

**Affiliations:** ^1^Department of Translational Medical Sciences, University of Naples Federico II, Naples, Italy; ^2^Department of Translational Medical Sciences, Center for Basic and Clinical Immunology Research, University of Naples Federico II, Naples, Italy; ^3^Clinic of Cardiovascular Diseases, IRCCS San Martino IST, Genova, Italy; ^4^Department of Medical Sciences and Public Health, University of Cagliari, Cagliari, Italy; ^5^Department of Molecular Biotechnology and Health Sciences, Molecular Biotechnology Center, University of Turin, Turin, Italy; ^6^Institute of Cardiology, Center of Excellence on Aging, Università degli Studi “G. d'Annunzio” Chieti – Pescara, Chieti, Italy; ^7^Department of Internal Medicine, Texas Heart Institute and Center for Cardiovascular Biology and Atherosclerosis Research, University of Texas Health Science Center, Houston, TX, United States; ^8^Section of Hygiene, Department of Public Health, University of Naples Federico II, Naples, Italy; ^9^Monaldi Hospital Pharmacy, Naples, Italy; ^10^Department of General Surgery and Medical-Surgery Specialities, University of Catania, Catania, Italy; ^11^U.O.C. Magnetic Resonance Imaging, Fondazione Toscana G. Monasterio C.N.R., Pisa, Italy; ^12^Division of Clinical and Experimental Cardiology, Department of Medicine and Pharmacology, Policlinico “G. Martino” University of Messina, Messina, Italy; ^13^Department of Clinical and Biological Sciences, University of Turin, Turin, Italy

**Keywords:** chemotherapy, HER-2 inhibitors, oxidative/nitrosative stress, vascular endothelial growth factor, tyrosine kinase inhibitors

## Abstract

Antineoplastic drugs can be associated with several side effects, including cardiovascular toxicity (CTX). Biochemical studies have identified multiple mechanisms of CTX. Chemoterapeutic agents can alter redox homeostasis by increasing the production of reactive oxygen species (ROS) and reactive nitrogen species RNS. Cellular sources of ROS/RNS are cardiomyocytes, endothelial cells, stromal and inflammatory cells in the heart. Mitochondria, peroxisomes and other subcellular components are central hubs that control redox homeostasis. Mitochondria are central targets for antineoplastic drug-induced CTX. Understanding the mechanisms of CTX is fundamental for effective cardioprotection, without compromising the efficacy of anticancer treatments. Type 1 CTX is associated with irreversible cardiac cell injury and is typically caused by anthracyclines and conventional chemotherapeutic agents. Type 2 CTX, associated with reversible myocardial dysfunction, is generally caused by biologicals and targeted drugs. Although oxidative/nitrosative reactions play a central role in CTX caused by different antineoplastic drugs, additional mechanisms involving directly and indirectly cardiomyocytes and inflammatory cells play a role in cardiovascular toxicities. Identification of cardiologic risk factors and an integrated approach using molecular, imaging, and clinical data may allow the selection of patients at risk of developing chemotherapy-related CTX. Although the last decade has witnessed intense research related to the molecular and biochemical mechanisms of CTX of antineoplastic drugs, experimental and clinical studies are urgently needed to balance safety and efficacy of novel cancer therapies.

## Introduction

**Figure 1:**
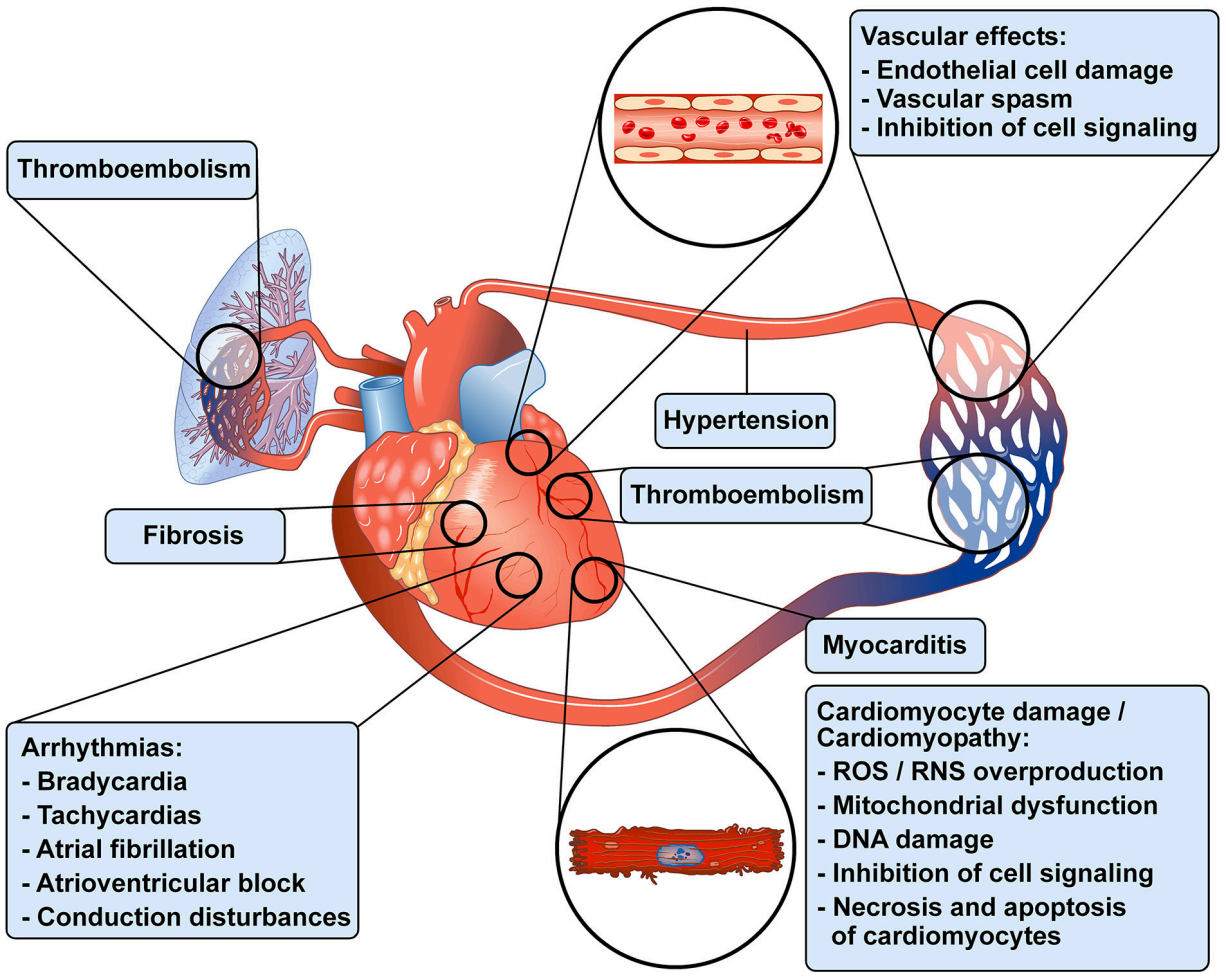
Schematic representation of some of the cardiovascular toxicities associated with antineoplastic drugs in patients with cancer. Modified with permission from Albini et al. (2010).

Antineoplastic treatments have improved overall survival and progression-free survival in the treatment of an increasing number of malignancies (Jemal et al., 2011). However, different antineoplastic drugs can cause a wide spectrum of cardiovascular (CV) toxicities (CTX), particularly in long-term cancer survivors (Oeffinger et al., 2006; Tocchetti et al., 2013; Moslehi and Deininger, 2015; Mercurio et al., 2016; Zamorano et al., 2016; Armenian et al., 2017). CTX include vasospastic and thromboembolic ischemia, hypertension, dysrhythmia, myocarditis and left ventricular (LV) dysfunction, leading to heart failure (Yeh and Bickford, 2009; Ky et al., 2013; Suter and Ewer, 2013; Zamorano et al., 2016). Figure 1 schematically illustrates the wide spectrum of cardiovascular toxicities associated with different antineoplastic drugs in patients with cancer. Anthracyclines (ANTs) can cause irreversible type 1 CTX through the production of reactive oxygen species (ROS) and reactive nitrogen species (RNS) (Ewer and Lenihan, 2008; Ewer and Ewer, 2010; Scott et al., 2011). Intracellular signaling inhibitors (e.g., tyrosine kinase inhibitors) block pathways that are main regulators of myocardial function, especially under conditions of cardiac stress, such as hypertension or hypertrophy (Suter and Ewer, 2013). The toxicity induced by biological drugs (e.g., trastuzumab) is often reversible, and has been classified as type 2 CTX (Ewer and Lippman, 2005; Ewer et al., 2005). However, these two forms of CTX may overlap. For example, trastuzumab, a monoclonal antibody anti-HER-2 (Shinkai et al., 1999), can cause irreversible LV dysfunction in patients previously treated with ANTs (Timolati et al., 2006; Suter and Ewer, 2013; Zamorano et al., 2016). More recently, immune myocarditis has entered as a novel challenge in the cardio-oncologic arena, due to a growing number of patients treated with immune checkpoint inhibitors (Swain and Vici) that unleash immune responses (Johnson et al., 2016; Varricchi et al., 2017d).

Here, we review the cellular and molecular mechanisms of CTX of antineoplastic drugs from a redox perspective, since plenty of evidence supports the importance of redox homeostasis for the maintainance of cardiovascular health, while anticancer drugs can disrupt such delicate balance in the myocardium and in the endothelium (Ferroni et al., 2011; Vincent et al., 2013; Zamorano et al., 2016).

## Oxidative and nitrosative stress in cardiovascular toxicity

ROS is a collective term that includes oxygen radicals, like superoxide (${\text{O}}_{2}^{-}$^•^) and hydroxyl radicals (OH^•^), and other non-radicals such as hydrogen peroxide (H_2_O_2_), singlet oxygen (^1^O_2_), etc. (Del Río, 2015). The term RNS includes radicals like nitric oxide (NO^•^) and nitric dioxide (NO_2_^•^), as well as non-radicals such as nitrous acid (HNO_2_) and dinitrogen tetroxide (N_2_O_4_), among others. Redox stress, resulting from overproduction of ROS and RNS, may directly or indirectly induce cardiac injury (Nediani et al., 2011; Willis and Patterson, 2013).

**Figure 2:**
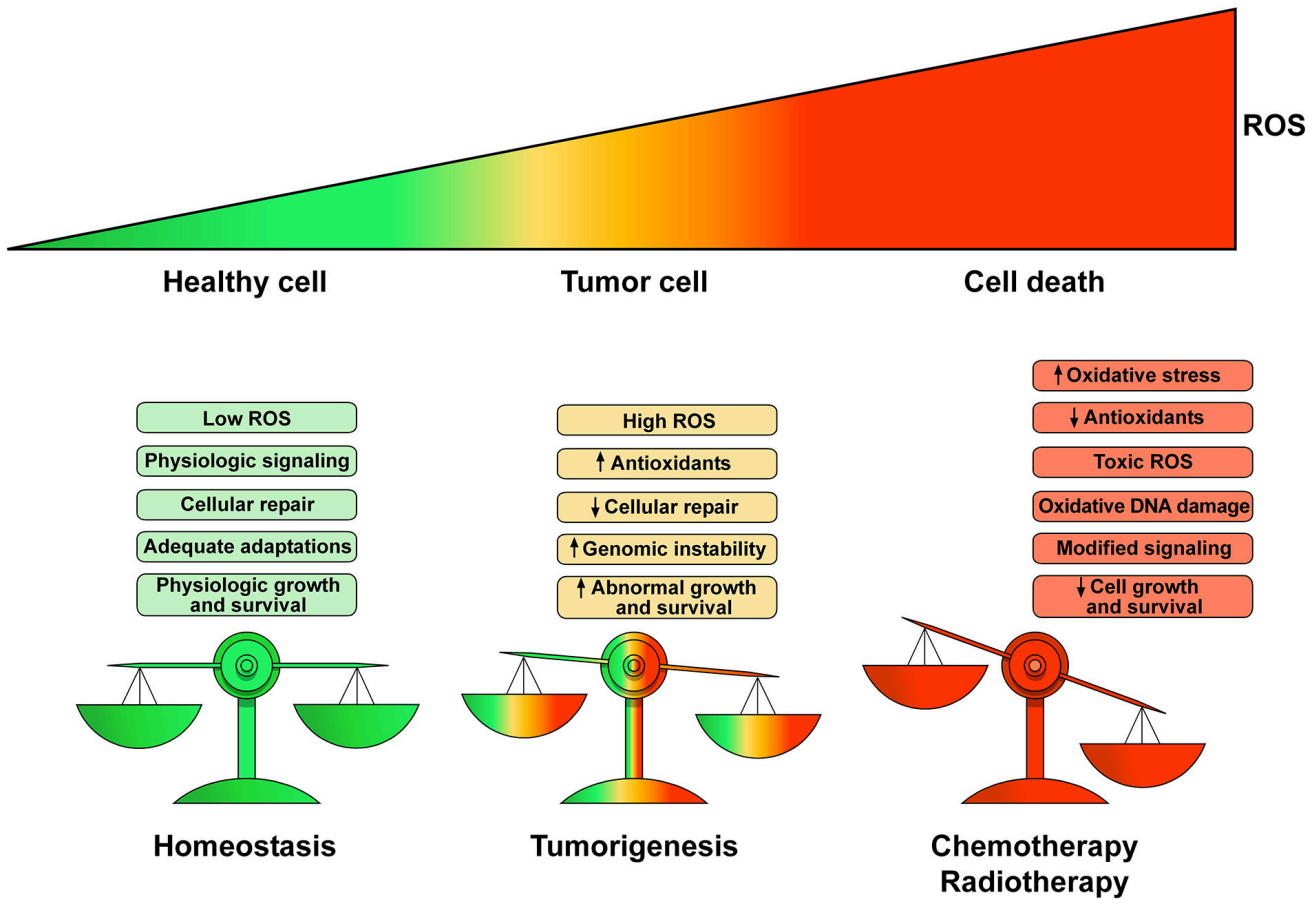
Schematic representation of the homeostatic role of ROS and their pathologic role in tumor growth and cell death. Low production of ROS and balanced antioxidant activity play a fundamental role in cellular signaling and repair resulting in controlled growth and survival. Proliferation of tumor cells yields elevated ROS concentrations enhancing cell survival and proliferation leading to DNA damage and genetic instability causing cell dysfunction. Chemotherapeutic agents and radiotherapy increase ROS production to toxic concentrations resulting in irreparable damage to the cell, inadequate adaptations and eventually cell death. The heart is particularly vulnerable to ROS/RNS injury because antioxidant resources are lower than other tissues. Modified with permission from Moloney and Cotter (2017).

Physiological levels of ROS and RNS are fundamental for the regulation of many cellular functions (Egea et al., 2017). For example, H_2_O_2_ is an endothelium-derived vasodilator of the coronary vessels (Saitoh et al., 2007). In pathological conditions (e.g., cancer growth) there is a deregulation of homeostatic control of ROS production leading to DNA damage, inhibition of cellular repair mechanisms and abnormal cell proliferation. ROS/RNS contribute to dysregulation of gene expression and genome stability, but also influence epigenetic pathways affecting the functions/expression of histone and DNA modifying enzymes (Mikhed et al., 2015; Niu et al., 2015). Several antineoplastic drugs induce CTX through an unbalanced generation of ROS/RNS, leading to the so-called oxidative/nitrosative stress. ROS/RNS imbalance derives from increased production or inactivation of endogenous antioxidant enzymes by antineoplastic drugs. Figure 2 schematically illustrates the transition from the homeostatic role of ROS/RNS in healthy subjects to the pathological role in cancer patients and during chemotherapy or radiotherapy.

The heart is particularly vulnerable to ROS/RNS injury because antioxidant resources are lower than other tissues (e.g., liver) (Minotti et al., 2004, 2010). High levels of ROS/RNS, by exhausting endogeneous antioxidant defenses, can hamper cellular signaling pathways in the CV system. Oxidative stress and low grade inflammation are interdependent processes implicated in cardiovascular diseases and cancer (Galdiero et al., 2016; Varricchi et al., 2017a). Tissue resident (e.g., macrophages, mast cells) and circulating inflammatory cells (e.g., neutrophils, monocytes) can also release ROS increasing oxidative stress (Varricchi et al., 2017a), interestingly ROS can initiate intracellular signaling increasing proinflammatory gene expression (Biswas, 2016).

High levels of ROS/RNS induce membrane lipid peroxidation and membrane damage, DNA damage and trigger death cell and apoptosis, leading to cardiomyocyte death and replacement by connective tissue, which results in irreversible cardiac damage (Li and Singal, 2000; Menna et al., 2008, 2012; Zang et al., 2012; Ky et al., 2013; Suter and Ewer, 2013; Hahn et al., 2014; Salvatorelli et al., 2015; Mercurio et al., 2016).

The major intracellular sources of ROS include the mitochondrial electron transport and the NADPH oxidase family (NOXs) (Lassègue and Griendling, 2010; Zhang et al., 2013). Mitochondria are key organelles for the regulation of redox signaling and redox homeostasis (Egea et al., 2017). Mitochondria function as a central hub that directly and indirectly controls redox homeostasis by hosting several redox-active complexes and enzymes that generate ROS and RNS. Mitochondria represent ≅35% of the myocyte volume and produce ≅90% of the cellular energy. Therefore, impairment of mitochondrial function is critical in cardiomyocytes (Pagliaro et al., 2011; Pagliaro and Penna, 2015; Tocchetti et al., 2015b). At present, the NOX family is composed of five isoforms (NOX1, NOX2, NOX3, NOX4, and NOX5). Cardiomyocytes (Varga et al., 2013) and macrophages (Moon et al., 2016) express NOX4. Mitochondrial and extramitochondrial NOX4 is a source of ROS and can be affected by anticancer drugs. Activated myocardial NOX2 produces ${\text{O}}_{2}^{•-}$, whereas NOX4 generates H_2_O_2_. Moreover, superoxide dismutases (SODs) convert ${\text{O}}_{2}^{•-}$ to H_2_O_2_. In mitochondria, H_2_O_2_ may be converted to O_2_ and H_2_O by catalase and by glutathione peroxidase (GPx). In the presence of iron complexes, these ROS may be converted to the more toxic OH^•^ within and outside mitochondria (Zhao et al., 2010; Pagliaro et al., 2011; Penna et al., 2014; Pagliaro and Penna, 2015; Tocchetti et al., 2015a). Interestingly, mitochondrial ROS are involved in the modulation of immune cells, including human neutrophils (Vorobjeva et al., 2017).

Peroxisomes, cytoplasmic organelles specialized for carrying out oxidative reactions, also play a role in ROS production/regulation in cardiomyocytes. Several substrates (i.e., amino acids, uric acid, and fatty acids) are broken down by oxidative reactions in peroxisomes. Fatty acid metabolism is very active in cardiomyocytes and peroxisomes are critical for processing long carbon chain fatty acids. The contribution of peroxisomes in the mechanism of CTX is largely unknown (Zanardelli et al., 2014).

Nitric oxide (NO) is a key regulator of cellular functions. It is a redox species with both oxidant and antioxidant properties (Takimoto and Kass, 2007; Pagliaro and Penna, 2015; Tocchetti et al., 2015a) produced produced from the metabolism of the amino acid, L-arginine by three isoforms of nitric oxide synthase (NOS): the endothelial (eNOS or NOS3) and neuronal (nNOS or NOS1) NOSs, constitutively expressed in cardiomyocytes, and the inducible NOS2 (iNOS), which is induced by pro-inflammatory mediators or by ischemia (Pagliaro and Penna, 2015; Tocchetti et al., 2015a). NO is also produced by other reactions termed “non-NOS” processes (Penna et al., 2014; Pagliaro and Penna, 2015). ROS can react with NO to form different RNS, thus amplifying the production of oxidant compounds, and NOS itself may produce ROS (Fogli et al., 2004; Penna et al., 2014; Pagliaro and Penna, 2015; Tocchetti et al., 2015b). NO together with RNS has an important role in mediating proteotoxic stress and modifications of mitochondrial activities, resulting in cytotoxicity and cell necrosis (Lala and Chakraborty, 2001). S-nitrosylation (SNO) is the covalent attachment of a NO moiety to a protein thiol group. SNO is a redox-dependent modification that exerts an antioxidant effect, shielding critical cysteine residues from oxidation and affecting protein functions (Penna et al., 2014; Pagliaro and Penna, 2015).

## Anthracyclines

The production of ROS/RNS is central in the CTX of several anti-cancer drugs. Some agents alter the activity of redox enzymes within and outside the mitochondria, including NOSs, respiratory complexes, the Krebs cycle, oxidative phosphorylation, and β-oxidation (Tocchetti et al., 2017). This impairment results in oxidative/nitrosative stress, a reduction in antioxidant capacity, and induction of cell death (Fogli et al., 2004; Albini et al., 2010; Mele et al., 2016a,b).

**Figure 3:**
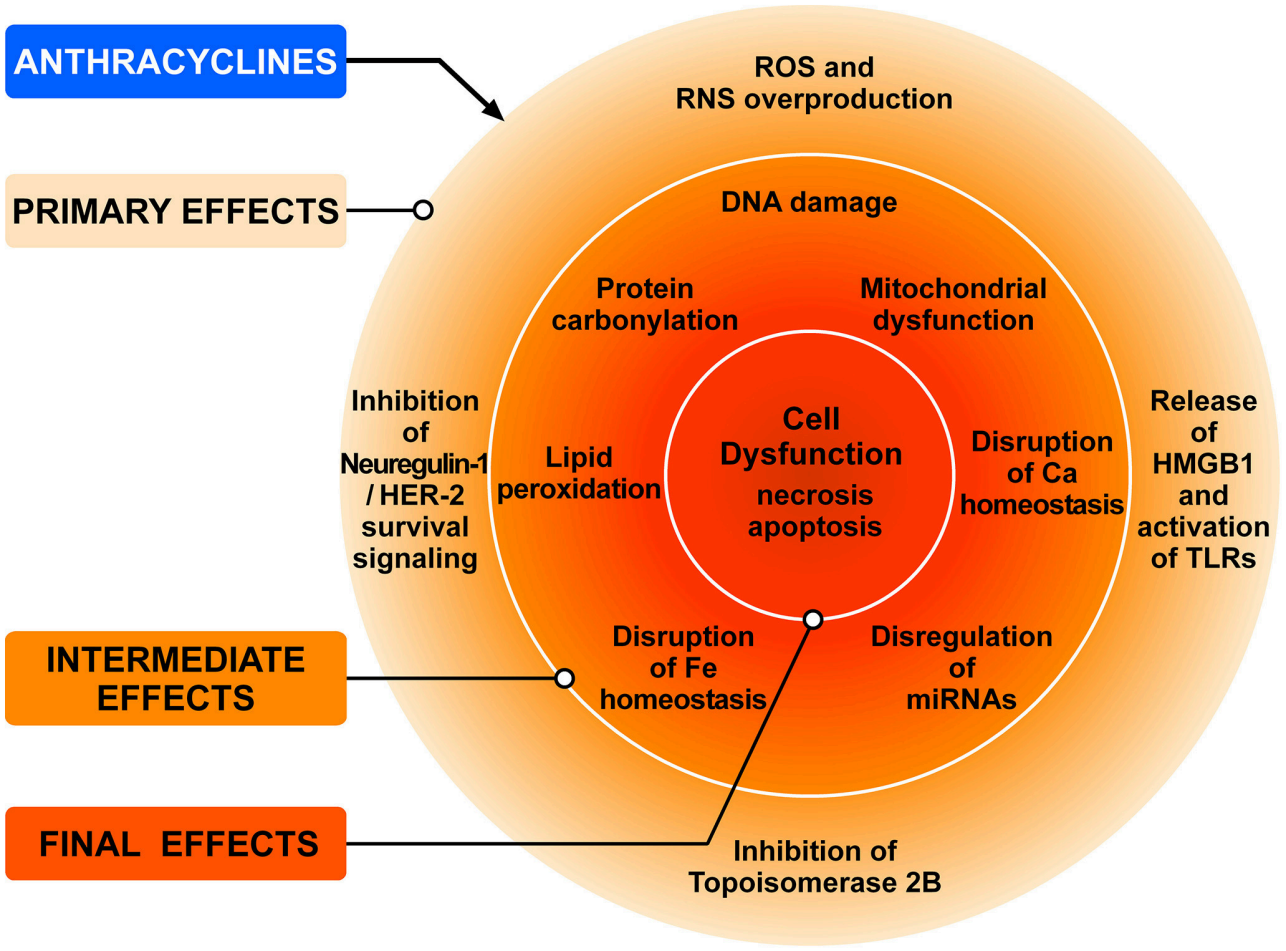
Schematic representation of the main mechanisms of anthracycline-induced injury to cardiac cells. The classic model of anthracycline (ANT) cardiotoxicity involves the generation of ROS by the quinone moiety common to all anthracyclines. ROS and RNS hyperproduction results in damage to DNA, protein carbonylation and lipid peroxidation leading to cellular dysfunction and cardiomyocyte death. ANTs can also bind and block the functions of both topoisomerases 2A (TOP2A) and 2B (TOP2B). Tumor cells express high levels of TOP2A, whereas TOP2B is ubiquitously expressed. Cardiomyocytes express TOP2B, but not TOP2A. ANTs form a complex with TOP2B inhibiting its enzymatic activity. Without functional TOP2B, DNA breaks accrue, leading to the activation of p53 tumor-suppressor protein, mitochondrial dysfunction, and the generation of ROS that result in cardiomyocyte death. Another mechanism underlying doxorubicin-dependent oxidative stress is linked to the ability of the drug to directly interfere with the activity of NADPH oxidase and nitric oxide synthase (NOS). Both NADPH oxidase and NOS can transfer electron from NADPH to doxorubicin, causing the formation of semiquinone doxorubicin (SQ-DOX). SQ-DOX in turn transfers electron to O_2_ and generates ${\text{O}}_{2}^{-}$. In the NOS compartment, ${\text{O}}_{2}^{-}$ can react with NO to form peroxynitrite (ONOO^−^), a powerful oxidant that can generate free radicals. An alternative mechanism by which ANTs exert their cardiotoxic effects is the inhibition of neuregulin-1 (NRG-1)-HER-2 in cardiomyocytes. Doxorubicin also induces necrosis of immune (i.e., macrophages) and cancer cells releasing HMGB1 which activates TLR-2 and TLR-4 in cardiomyocytes and inflammatory cells inducing the release of proinflammatory cytokines. These primary effects induce a plethora of secondary effects in cardiomyocytes (e.g., DNA damage, lipid peroxidation, mitochondrial dysfunction, etc.,) which result in cell dysfunction and death. Modified with permission from Tocchetti et al. (2017).

ANTs (doxorubicin, epirubicin and daunorubicin), widely used as anticancer agents, are recognized as prototype of type 1 CTX since the 1960s (Tan et al., 1967). ANTs can induce LV dysfunction, leading to HF in up to 9% of patients (Cardinale et al., 2015). ANT can cause CTX *via* a series of many cellular and molecular mechanisms (Zhang et al., 2012; Zamorano et al., 2016). Figure 3 schematically illustrates the complex interplay of the major mechanisms by which ANTs can induce injury to cardiac cells. The administration of ANTs can alter redox homeostasis in cardiomyocytes and tissue resident (e.g., fibroblasts, endothelial cells, mast cells, macrophages) and circulating inflammatory cells (e.g., neutrophils, eosinophils) in the heart by producing ROS and RNS (Pagliaro and Penna, 2015; Ghigo et al., 2016; Tocchetti et al., 2017).

A basic mechanism by which ANTs can cause CTX is the interaction with topoisomerase 2 (TOP2) A and -B highly expressed in cardiomyocytes (Lyu et al., 2007). The former is present in rapidly dividing cells, such as cancer cells, and forms the ternary TOP2-doxorubicin-DNA complex, inducing cell apoptosis. TOP2B, highly expressed in human cardiomyocytes, forms the TOP2B-doxorubicin-DNA complex, which causes DNA damage leading to cell apoptosis. The tumor suppressor protein p53, a pivotal enzyme for activating DNA repair proteins, can cause mitochondrial dysfunction and metabolic failure (Sawyer, 2013). The metabolic alterations caused by doxorubicin-activated p53 damage mitochondria in the cardiomyocytes, result in enhanced ROS/RNS generation and ultimately cell death. Collectively, these results indicate that oxidative reactions play a central role in ANT-induced LV dysfunction. Therefore, drugs that interfere with molecules involved in heart metabolism (e.g., p53) may represent a potential approach in limiting LV dysfunction (Sawyer, 2013; Mercurio et al., 2016).

Besides directly damaging cardiomyocytes, doxorubicin induces apoptosis of immune (e.g., macrophages) and cancer cells releasing high mobility group box 1 (HMGB1) which, in turn, triggers toll-like receptor (TLR)-2 and-4 (Ma et al., 2012; Yao et al., 2012). TLR-2 and TLR-4 are found in cardiomyocytes and inflammatory cells and their engagement induces the release of proinflammatory cytokines (i.e., IL-6, IL-1β, TNF-α). Overall, these findings emphasize the contribution of TLRs in mediating ANT-induces inflammation and CTX and envisage the possibility of targeting this pathway for therapeutic purposes.

A better characterization of the multiple molecular mechanisms of ANT-related toxicity of blood vessels and cardiomyocytes appears fundamental to select the best approach to prevent and treat CTX (Van Cutsem et al., 2002; Scott et al., 2011; Madonna et al., 2015a,b; Cadeddu et al., 2016).

As mentioned in a previous section, mitochondrial ROS (mtROS) represent a prominent source (≅80%) of ROS, expecially in the heart (Russell and Cotter, 2015). mtROS play a pivotal role in ANT-induced CTX (Minotti et al., 2004, 2010). Doxorubicin binds with high affinity to the mitochondrial phospholipid cardiolipin, inhibits its function, stimulates ROS/RNS production, inhibits oxidative phosphorylation, and causes mitochondrial DNA damage (Pereira et al., 2016). ANTs also cause mitochondrial calcium accumulation, leading to mitochondrial injury (Pereira, Pereira et al., 2016). ANTs can also affect cardiac progenitor cells following myocardial injury (Huang et al., 2010; Oliveira et al., 2013).

The production of ROS is a central event in ANT-induced CTX. ROS are effectors of membrane lipid peroxidation, irreversible damage, and myocyte replacement by connective tissue (Menna et al., 2008, 2012; Zhang et al., 2012; Ky et al., 2013; Suter and Ewer, 2013; Salvatorelli et al., 2015). ROS generated by ANTs affect mitochondrial enzymes, NOSs, NAD(P)H oxidases, and catalase, leading to oxidative stress and cell injury. ANTs are metabolized to unstable compounds (such as doxorubicin-semiquinone), which react with O_2_, producing H_2_O_2_ and ${\text{O}}_{2}^{•-}$.

ANTs chelate free intracellular iron, forming iron-doxorubicin complexes. ANTs also interfere with iron-transporting and -binding proteins (Gammella et al., 2014; Ghigo et al., 2016). Ardehali and collaborators found that doxorubicin impairs a mitochondrial iron exporter with consequent iron accumulation and subsequent ROS generation (Ichikawa et al., 2014). Cardiac dysfunction following ANT treatment is associated with high mitochondrial iron levels compared with normal hearts (Ichikawa et al., 2014). Collectively, these findings indicate that oxidative stress and mitochondrial iron accumulation play a key role in ANT-induced CTX.

ANTs interact with cardiolipin leading to concentration of the drug in mitochondrial membrane phospholipids (Goormaghtigh et al., 1990). In mitochondria, the drug exerts adverse effects (e.g., ROS generation, inhibition of oxidative phosphorylation, and mitochondrial DNA damage). ROS cause peroxidation of cardiolipin, which induces the release of mitochondrial factors, such as cytochrome c, which in turn triggers cardiolipin peroxidation. This cycle exacerbates ANT-induced injury. NO inhibits both the peroxidase activity of cytochrome c and cardiolipin oxidation. NO, which possesses antioxidant properties, may counteract the toxic effects of ANTs (Vlasova et al., 2006; Gonzalvez and Gottlieb, 2007; Pointon et al., 2010).

Enzymes located outside the mitochondria also able to produce ROS. A nonexhaustive list includes NADPH oxidases (NOXs), xanthine oxidase (XO), and monoamine oxidase. Xanthine oxidase and NADPH, may be targeted by ANTs. Doxorubicin deoxyaglycone can be obtained by a reduction process and accumulates in membranes, altering the function of NADH dehydrogenase in mitochondria or the NOXs in the plasma membrane (Thorn et al., 2011). Among other mechanisms involved in cardiotoxicity caused by ANTs, recent studies have highlighted the role of altered myocardial energetics, expressed by a lower phosphocreatine/adenosine triphosphate (ATP) ratio, which precedes LV dysfunction (Maslov et al., 2010). Indeed, ANTs can oxidize sulfhydryl groups of creatine kinase (CK), reducing its function, thus impairing myocardial energetics (Maslov et al., 2010), hence causing LV dysfunction. More studies on such an interesting mechanism could be helpful in order to identify new protective therapeutic strategies. Indeed, overexpression of myofibrillar CK in mice with HF induced by transverse aortic constriction increased heart function (Gupta et al., 2012) supporting a role for CK in HF prevention and treatment. Accordingly, the same group demonstrated that CK overexpression also ameliorated myocardial energetics, contractile function, and survival in murine anthracyclines cardiotoxicity (Gupta et al., 2013). These results provide novel strategies for limitation of anthracycline-related cardiotoxicity.

ANTs are also able to alter cardiac energy metabolism by lowering the level of 5′ AMP-activated protein kinase (AMPK, activated in the response to energy stress) and phosphorylation of anti-acetyl-CoA carboxylase, leading to impairment of fatty acid oxidation (Tokarska-Schlattner et al., 2005). The mechanisms underlying inhibition of AMPK need to be fur-ther elucidated (Mercurio et al., 2016).

Importantly, along with ANTs (Menna et al., 2012; Sawyer, 2013; Sterba et al., 2013; Ghigo et al., 2016), redox abnormalities are central in the pathophysiology of cardiotoxicity caused by other anticancer drugs, among which are new biologic antineoplastic agents, such as intracellular signaling inhibitors, that are increasingly being used (Tocchetti et al., 2017). Such agents may cause cardiotoxicity, since they block pathways important for the modulation of myocardial function, especially under conditions of cardiac stress, such as hypertension or hypertrophy (Suter and Ewer, 2013), with mechanisms of action that often involve redox dysregulation as well.

## Antimetabolites

Fluoropyrimidines [i.e., *5*-fluorouracil (5-FU), capecitabine, and gemcitabine] are used in the treatment of several tumors. 5-FU administered intravenously has a short half-life, but active metabolites concentrate in cardiac and cancer cells, resulting in a prolonged exposure to the drug (Kosmas et al., 2008; Miura et al., 2010; Lestuzzi et al., 2011). Capecitabine is converted into its active form preferentially within tumors (Ng et al., 2005; Aprile et al., 2009; Khan et al., 2014; Petrelli et al., 2016). 5-FU and its main metabolite can induce CTX after few days of treatment (Jensen and Sorensen, 2006; Jensen and Sørensen, 2012). The enzyme involved in the conversion of capecitabine to 5-FU is expressed in both atherosclerotic plaques and cancer cells, explaining the CTX in patients with coronary artery disease. The incidence of CTX caused by 5-FU ranges from 0 to 35%, with a mortality rate between 2 and 13%. Myocardial ischemia is the strongest risk factors for fluoropyrimidine-induced CTX (Koca et al., 2011; Polk et al., 2013, 2014). Silent ischemia due to cardiac stress test has been reported in 6–7% of 5-FU-treated patients (Lestuzzi et al., 2014). The mechanisms involved in the CTX of 5-FU and its metabolites involve inhibition of NO (Cianci et al., 2003; Shoemaker et al., 2004), enhanced generation of ROS/RNS (Lamberti et al., 2014), higher endothelial thrombogenicity (Kalam and Marwick, 2013) and senescence (Altieri et al., 2017), and DNA and RNA damage. 5-FU can induce oxidative stress in cardiomyocytes and endothelial cells. This drug causes eNOS dysregulation, endothelin 1 upregulation and the activation of protein kinase C. These effects lead to endothelium-dependent and -independent vasoconstriction, and eventually to coronary spasm (Alter et al., 2006; Sorrentino et al., 2012).

## Her-2 inhibitors

Epidermal growth factor receptor 2 (ErbB2) (also called HER-2), ErbB1, ErbB3, and ErbB4 are members of the human epidermal growth factor receptor family. When activated by their ligands, these transmembrane receptors homodimerize or heterodimerize and are phosphorylated, initiating several cellular responses (Force et al., 2007). HER-2, present on human heart and overexpressed in approximately 30% of breast cancers, can interact with HER-1 and HER-3, independently from ligand stimulation, thus triggering signaling pathways that stimulate tumor growth (Slamon et al., 1987). Trastuzumab, a humanized mAb that binds the extracellular domain IV of HER-2 (Force et al., 2007; Suter et al., 2007), can cause type 2 CTX (Ewer and Lippman, 2005; Ewer et al., 2005) in approximately 30% of patients when combined with ANTs (Slamon et al., 2001; Suter et al., 2007; De Keulenaer et al., 2010).

Several oral small molecules inhibiting tyrosine kinase (TK) associated with HER are clinically used or under development (De Keulenaer et al., 2010; Ades et al., 2014). Lapatinib and neratinib are novel HER-2/HER-4 TK inhibitors undergoing clinical development in HER-2^+^ breast cancer. Their cardiac safety data show a favorable profile (Ades et al., 2014). Several clinical trials have demonstrated that lapatinib is less toxic than trastuzumab (Ades et al., 2014). Pertuzumab is a humanized mAb blocking domain II of the extracellular part of HER-2, thus stopping HER-2/HER-3 homo-heterodimeration. Several clinical trials have assessed the cardiac toxicity of pertuzumab (Bowles et al., 2012; Molinaro et al., 2015). Pertuzumab causes a modest (≅10%) reduction of LVSD in patients with HER-2^+^ breast cancer (Baselga et al., 2012; Gianni et al., 2012; Swain et al., 2013).

Importantly, in breast cancer treatment, the co-administration of trastuzumab with ANTs enhances the latter's toxicity and is now avoided. In fact, anti-HER-2 mAbs block the protective mechanisms of HER-2, exhacerbating the oxidative damage caused by doxorubicin (Ewer and Ewer, 2010). Indeed, redox mechanisms have also been advocated for the neuregulin/ErbB2 pathway. This pathway can modulate the increase in ROS caused by doxorubicin in animal models (Timolati et al., 2006), suggesting that cardiotoxicity from ErbB2 blockade can also involve a dysregulation of redox homeostasis (Gordon et al., 2009; Mercurio et al., 2016).

**Figure 4:**
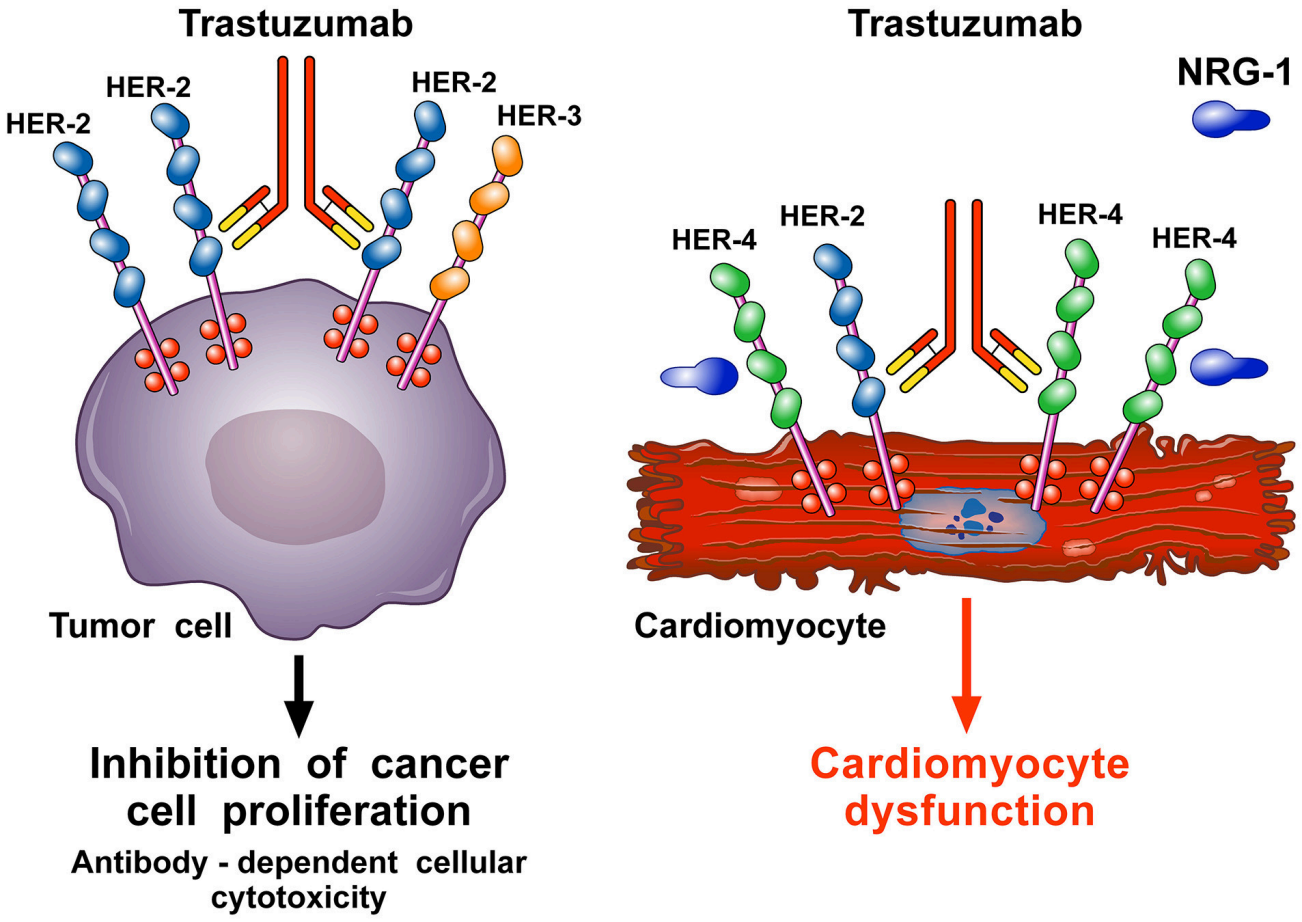
Schematic representation of the mechanism of action of trastuzumab and pathogenesis of its cardiotoxicity. Trastuzumab is a mAb that binds the extracellular domain IV of HER-2. It is used to treat breast cancer patients (≅30%) in which HER-2 is overexpressed and spontaneously homodimerizes or forms heterodimers with other HER receptors, especially HER-3. This ligand-independent activation of HER-2 promotes proliferation and survival of tumor cells. Trastuzumab blocks the interaction HER-2/HER-3 and downstream signaling halting the growth of tumor cells. Moreover, trastuzumab induces the antibody-dependent immune cell-mediated cytotoxicity of cancer cells **(left side)**. In the heart, neuregulin-1 (NRG-1) triggers HER-4/HER-4 homodimerization and HER-4/HER-2 heterodimerization on cardiomyocytes to induce protective pathways in response to stress. Blockade of cardiac HER-2 by trastuzumab results in the disruption of NRG-1-dependent signaling and consequently in alterations of structure and functions that cause cardiomyocyte death **(right side)**.

In the heart, endothelial cells release neoregulin 1 (NRG-1), especially the NRG-1β isoform (Lim et al., 2015), which triggers HER-4/HER-4 homodimerization and HER-4/HER-2 heterodimerization on cardiomyocytes to activate protective pathways in response to stress (De Keulenaer et al., 2010; Odiete et al., 2012; Lim et al., 2015; Figure 4). The HER-2 pathway mediates cell survival and possibly regeneration (D'Uva et al., 2015) and is stimulated when the heart experiences stress, including hypertension (de Korte et al., 2007; Ewer and Ewer, 2010) and ANT therapy (Gabrielson et al., 2007). Anti-HER-2 agents interfere with the NRG-1/HER-4/HER-2 axis and can cause cardiomyocyte damage. This hypothesis is corroborated by ErbB2 KO-mice that present with LV dilation and increased susceptibility to ANT-induced cardiac damage (Crone et al., 2002; Ozcelik et al., 2002), supporting a fundamental role of HER-2 in the heart. Conversely, cardiac ErbB2 overexpressed mice exhibited reduced levels of ROS in mitochondria, with lower ROS levels and less cell death after treatment of neonatal cardiomyocytes isolated from ErbB2 (Bosch et al., 2013) hearts with anthracyclines. This was due to higher levels of glutathione peroxidase 1 (GPx1) protein and GPx activity, with increased levels of two known GPx activators, c-Abl and Arg (Belmonte et al., 2015; Tocchetti et al., 2017).

HER-2 and HER-4 receptor expression and activation/phosphorylation are lower in failing human myocardium, a condition characterized by increased oxidative stress (Rohrbach et al., 1999). Dogs with HF showed increased phosphorylation of ErbB4 and ErbB2 (Doggen et al., 2009). NRG-1 expression is enhanced in HF (Rohrbach et al., 1999; Doggen et al., 2009). Collectively, these results indicate that NRG-1/HER-4/HER-2 activity is involved in the pathophysiology of HF (Mercurio et al., 2016). (Mercurio et al., 2016). Importantly, NRG-1 exerts a lusitropic effect on isolated cardiac muscle preparations via a NO-dependent mechanism (Lemmens et al., 2004): this requires a functional NO synthase, with preserved NO bioavailability, a condition which can be hampered by the increased oxidative stress in HF (Nediani et al., 2011; Arcaro et al., 2016).

Based on cardioprotective properties of NRG-1 *via* HER-4/HER-2, the neuregulin-HER pathway is currently being assessed in clinical studies for HF treatment (Galindo et al., 2014a,b). NRG-1β increases LV function and reduces cardiac dimensions in experimental failing hearts (Liu et al., 2006; Li et al., 2011; Galindo et al., 2014a,b; Mercurio et al., 2016). NRG-1 also inhibits cardiac fibroblasts and prevents fibrosis (Galindo et al., 2014a,b). NRG-1 administration after myocardial infarction is able to blunt remodeling of the damaged heart (Liu et al., 2006; Galindo et al., 2014a,b). Clinical trials have shown that NRG-1 is well-tolerated and ameliorates heart dimensions and LVEF up to 3 months after treatment (Gao et al., 2010; Jabbour et al., 2011). However, NRG-1 may be a growth factor for cancer cells, and further studies are necessary to assess the safety and efficacy of NRG-1 in HF (Lim et al., 2015; Mercurio et al., 2016).

## Inhibitors of vascular endothelial growth factor (VEGF) signaling

Vascular endothelial growth factors (VEGF-A, VEGF-B, VEGF-C, VEGF-D. and PlGF) activate specific tyrosine kinase (TK) receptors (VEGFR-1, VEGFR-2, and VEGFR-3) on blood endothelial cells (Loffredo et al., 2016; Staiano et al., 2016) and on endothelial colony forming cells (Dragoni et al., 2011) and have a major role in myocardial angiogenesis at rest and in pressure-overload hearts (Oka et al., 2014). Inhibitors of VEGF signaling (i.e., mAbs anti-VEGF-A and “specific” TK inhibitors) are used for the treatment of several malignancies (Hurwitz et al., 2004; Sandler et al., 2006). VEGFs also regulate several myocardial functions and the integrity of coronary and systemic blood vessels (Folkman, 2007; Eschenhagen et al., 2011; Curigliano et al., 2012; Tocchetti et al., 2013; Marone and Granata, 2014), hence, not surprisingly, beside fighting cancer proliferation by inhibiting angiogenesis, VEGF antagonists may produce different forms of CTX, mainly hypertension, thromboembolism, LV dysfunction, and HF (Gressett and Shah, 2009; Nazer et al., 2011; Welti et al., 2013).

Bevacizumab (anti-VEGF mAb), sunitinib and sorafenib (TK inhibitors: TKIs) are used for the treatment of different types of cancer (Hurwitz et al., 2004; Sandler et al., 2006). Bevacizumab can induce hypertension and cardiac dysfunction in 1–3% patients undergoing chemotherapy (Miller et al., 2005). Regorafenib is a multi-target TKI that inhibits VEGFR1, endothelial-specific receptor tyrosine kinase (trk2), PDGFR, fibroblast growth factor receptor (FGFR), KIT, and RET. Regorafenib, used in therapeutic protocolos for gastrointestinal tumors, can induce hypertension (Brinda et al., 2016) and less frequently cardiac ischemia and myocardial infarction (Bronte et al., 2015). Treatment with pazopanib and axitinib (inhibitors of VEGFRs, PDGFRA and B, and KIT) can lead to hypertension (Motzer et al., 2013). 40% of patients treated with axitinib can experience hypertension (Hutson et al., 2013). Novel anti-angiogenic drugs such as cediranib, vatalanib and nintedanib also exhibit a potential risk of hypertension and HF (Goss et al., 2010; Van Cutsem et al., 2011; Reck et al., 2014).

Sunitinib and sorafenib are not selective TKIs and inhibit several kinases other than VEGFR (Cheng and Force, 2010). Sunitinib inhibits more than 30 TKs, including platelet-derived growth factor receptor (PDGFR), KIT, and colony-stimulating factor 1 receptor (CSF1R) (Force et al., 2007; Cheng and Force, 2010; Hasinoff and Patel, 2010). All these kinases are regulators of CV functions (Lévy, 2006; Anisimov et al., 2009). Up to 28% of patients can develop cardiac dysfunction from sunitinib (Chu et al., 2007; Motzer et al., 2007; Khakoo et al., 2008; Telli et al., 2008). The CTX induced by sunitinib is also due to interference with ribosomal S6 kinase (Tokarska-Schlattner et al., 2005) that then triggers apoptosis (Force et al., 2007; Kerkela et al., 2009). Sunitinib prolongs opening of the mitochondrial permeability transition pore (mPTP) and mitochondrial swelling in myocytes from heart subjected to pressure overload (Chu et al., 2007). Also, treatment of different myocardial preparations with sunitinib produces a dose-dependent negative inotropic effect, paralleled by a decline in intracellular Ca^2+^ and increase of ROS production (Rainer et al., 2012; Tocchetti et al., 2013). Interestingly, our preliminary data show that CK might play a role in the regulation of sunitinib cardiac effects (Tocchetti et al., 2015b). In addition, sutinitib can harm pericytes in cardiac vessels (Chintalgattu et al., 2013). Sorafenib inhibits at least 15 kinases, including the VEGFR, PDGFR, and KIT (Force et al., 2007; Cheng and Force, 2010; Tocchetti et al., 2013).

In conclusion, cardiac dysfunction can be induced by many mechanisms in patients treated with mAbs anti-VEGF and TKIs including alterations of mitochontrial function and energy production with increase in ROS generation, as well as induction of arterial hypertension (Mourad and Levy, 2011). Bevacizumab and sunitinib can cause hypertension because of functional (inactivation of endothelial NO synthase and production of vasoconstrictors such as endothelin-1) and anatomic modifications, bringing to vasoconstriction and to an increase in peripheral vascular resistance (Ku et al., 1993; Mourad and Levy, 2011; Nazer et al., 2011; Hahn et al., 2014). Arterial and venous thrombosis is due to reduction of NO synthesis, endothelial dysfunction, and plaque instability.

## Antioxidant properties of cardiovascular drugs: a useful tool for the protection from cardiotoxicity of antineoplastic drugs

It has been suggested that drugs with antioxidant properties can prevent CTX induced by an increase in ROS (Swain et al., 1997; Li and Singal, 2000; Spallarossa et al., 2004; Cadeddu et al., 2010; Lipshultz et al., 2012; Dessí et al., 2013; Broeyer et al., 2014). Dexrazoxane, an iron-chelating drug, is a cardioprotective agent approved by the FDA for ANT-induced CTX. It is a pro-drug that enters the cardiomyocyte, is rapidly metabolized into its active form, and inhibits the formation of ANT-iron complexes and the production of ROS (Simunek et al., 2009). Its efficacy in several types of tumors has been demonstrated in clinical trials and two pooled analyses (Swain et al., 1997; Seymour et al., 1999; Swain and Vici, 2004; Lipshultz et al., 2012). Other iron chelators have not shown any cardioprotective effect suggesting that dexrazoxane exerts its effects by means of additional protective mechanisms (Simunek et al., 2009). Dexrazoxane changes the Top2β configuration preventing its interface with ANTs, thereby impeding the formation of the Top2-DNA complexes (Lyu et al., 2007; Lencova-Popelova et al., 2016). Stěrba and coworkers have shown that the cardioprotective effects of dexrazoxane are due to its interaction with Top2-β, rather than to its iron chelating activity (Sterba et al., 2013). Derivatives of dexrazoxane lacking effects on Top2β were found not to be protective in models of ANT-induced CTX (Martin et al., 2009; Tocchetti et al., 2017) suggesting the relevance of Top2β in the cardioprotective mechanism.

## Antioxidant properties of beta blockers: beyond the antiadrenergic effects

β-blockers are cornerstone treatments for patients with low LVEF (Ponikowski et al., 2016), and there is evidence to encourage their use in asymptomatic ANT-related LV dysfunction (Curigliano et al., 2012; Cadeddu et al., 2016). The rationale for β blocker utilization in ANT-induced CTX is based on clinical and experimental results. Alterations of β-adrenergic receptor (β-AR) signaling are present in LV dysfunction caused by ANTs and in other types of dilated cardiomyopathies (Fu et al., 1994). Furthermore, a positive effect of β-AR blockage in reducing oxidative stress and myocardial calcium overload (Nakamura et al., 2002; Asanuma et al., 2004) has been shown in experimental models. New-generation β blockers (i.e., carvedilol and nebivolol) have been taken into consideration for their cardioprotective properties. Carvedilol, a non-selective β- and α1-AR antagonist with strong antioxidant properties, was compared to atenolol, a β blocker devoid of antioxidant properties. Only carvedidol conferred protection from ANT-induced LV-dysfunction and such effect has been attributed to its antioxidant properties rather than to the β-AR blocking action (Matsui et al., 1999). Carvedilol inhibits ANT-induced ROS release, cardiomyocyte apoptosis (Spallarossa et al., 2004), and mitochondrial alterations (Santos et al., 2002). In a small clinical trial evaluating the cardioprotective effect of carvedilol in patients treated with ANTs a reduced incidence of LV dysfunction was reported (Kalay et al., 2006). More studies are needed in order to confirm this cardioprotective effect.

In an experimental model of ANT-induced CTX, nebivolol, a cardio-selective β blocker with limited vasodilating properties, improved LV function, while enhancing NO levels and lowering oxidative stress (de Nigris et al., 2008; Tocchetti et al., 2015a). In a small clinical trial the prophylactic use of nebivolol in patients undergoing ANT-based treatments was associated with lower incidence of LV dilatation and systolic dysfunction in the nebivolol group compared to the placebo group (Kaya et al., 2013).

Interestingly, β blockers have been associated with reduced risk of cardiac dysfunction in patients on trastuzumab, ANTs, or both (Seicean et al., 2013). More recently, β blockers such as bisoprolol (Pituskin et al., 2017) and metoprolol have not shown promising results in the prevention of trastuzumab-induced LV dysfunction, suggesting that blockade of β1 alone is not cardioprotective (Gulati et al., 2016). This supports the use of non-selective β1 and β2 blockers (Sysa-Shah et al., 2016).

## The redox role of renin-angiotensin-aldosterone system antagonists

The renin-angiotensin-aldosterone system (RAAS) is a key player in ANT-induced CTX (Arnolda et al., 1985). Angiotensin-converting enzyme inhibitors (ACE-Is) and angiotensin II receptor blockers (ARBs) can reduce the progression of heart dysfunction and prevent HF in high-risk patients (Ponikowski et al., 2016). Experimental studies have shown the efficacy of ACE-Is in fighting ANT-induced CTX (Abd El-Aziz et al., 2001; Boucek et al., 2003). ACE-Is can confer protection from ANT-related CTX by reducing ROS damage, intracellular calcium overload and fibrosis, and by enhancing mitochondrial respiration and cardiomyocyte metabolism (Abd El-Aziz et al., 2001; Boucek et al., 2003). Enalapril, captopril, and lisinopril can improve acute and chronic ANT-induced cardiotoxicity in experimental models (Abd El-Aziz et al., 2001). In ANTs-treated patients, enalapril reduced the incidence of LV dysfunction compared to placebo (Cardinale et al., 2015). Candesartan modulates experimental cardiotoxicity induced by ANTs (Soga et al., 2006). Pre- and post-treatment with telmisartan protects against acute doxorubicin-induced LV dysfunction in rats (Iqbal et al., 2008). Telmisartan affects the bioavailability of NO and inhibits the production of interleukin-6 (IL-6) and tumor necrosis factor-α (TNF-α) (Yamagishi and Takeuchi, 2005). In a small prospective study, telmisartan blunted subclinical cardiotoxic effects of epirubicin (EPI) (Cadeddu et al., 2010). Telmisartan reversed early EPI-induced myocardial dysfunction and maintained a normal systolic function up to the 18-month follow-up (Dessí et al., 2011, 2013). Valsartan exerted a cardioprotective effect in patients treated with ANTs (Nakamae et al., 2005).

The combination of ACE-Is (enalapril) and β blockers (carvedidol) seems to be beneficial in treating ANT-induced CTX (Bosch et al., 2013). Several clinical trials have evaluated the role of ACE-Is and ARBs as cardiopreventive agents in patients undergoing chemotherapy (Lim et al., 2015; Molinaro et al., 2015). A recent meta-analysis showed that the prophylactic administration of ACE-Is and ARBs in patients treated with ANTs reduced the risk of developing CTX compared with placebo (Kalam and Marwick, 2013). Unfortunately, recent studies have failed to show promising results about prevention of cardiotoxicity with beta blockers or ACE-Is or ARBs (Boekhout et al., 2016; Gulati et al., 2016; Pituskin et al., 2017).

Non-dihydropyridine calcium channel blockers are not indicated in patients with anti-angiogenic drug-induced hypertension, due to the pharmacokinetic interaction of sorafenib and sunitinib with CYP3A4 (Maitland et al., 2010; Cadeddu et al., 2016). Experimental and clinical studies should evaluate the safety and efficacy of the combination of ACE-Is and β blockers in preventing sunitinib-induced CTX.

## Experimental antioxidant drugs in cardioprotection against cardiotoxic effects of anthracyclines

Several drugs (e.g., ranolazine, statins, and phosphodiesterase-5 inhibitors) have been assessed in counteracting ANT-induced CTX. The efficacy of different statins in preventing ANT-induced CTX is so far unproven, due to controversial data. Statins (i.e., lovastatin and fluvastatin) were cardioprotective in cellular studies performed on proliferating H9c2 cell line, but not on cardiomyocytes (Riad et al., 2009; Huelsenbeck et al., 2011). Lovastatin did not modify LV dysfunction induced by doxorubicin (Henninger et al., 2015). Small clinical studies have reported protective/marginal effects of statins in patients treated with ANTs (Seicean et al., 2012; Chotenimitkhun et al., 2015). Hence, several experimental models of cardiac dysfunction have suggested a cardioprotective effect with ranolazine (Sabbah et al., 2002; Rastogi et al., 2008; Coppini et al., 2013, 2017). Ranolazine can preserve cardiac function in mice treated with ANTs by reducing oxidative stress (Tocchetti et al., 2014; Cappetta et al., 2017). Ranolazine can prevent calcium overload and the occurrence of oxidative damage by suppressing ROS production (Kohlhaas et al., 2010). Although the INTERACT study indicated that ranolazine was a promising agent for the prevention of DOX-induced cardiotoxicity, more studies are needed to confirm such evidence (Minotti, 2013).

Sildenafil, a phosphodiesterase-5 inhibitor, seems to protect from ANT-induced cardiac dysfunction by opening mitochondrial K_ATP_ channels, preserving mitochondrial membrane potential and myofibrillar integrity, and preventing cardiomyocyte apoptosis (Fisher et al., 2005). Tadalafil blunted ANT-induced LV dysfunction through NO-mediated rises in cGMP levels (Koka et al., 2010; Jin et al., 2013).

Hydrogen sulfide (H_2_S), a redox compound, also attracted the interest of cardio-oncologists. Cystathionine gamma-lyase, a key enzyme in the synthesis of H_2_S, is involved in ANT-induced CTX in cardiomyocytes and exogenous H_2_S has been shown to protect against CTX (Papapetropoulos et al., 2015; Cadeddu et al., 2016; Mele et al., 2016b). Further experimental research and randomized trials will be needed to assess the safety and efficacy of H_2_S.

Experimental data show that VEGF-B favors coronary artheriogenesis, physiological cardiac hyperthrophy, and resistance to ischemia (Bry et al., 2010; Kivelä et al., 2014). Furthermore, VEGF-B has been proposed as a candidate for the therapy of dilated cardiomyophaty (Kivelä et al., 2014; Woitek et al., 2015). There is preliminary evidence that VEGF-B gene therapy can inhibit doxorubicin-induced CTX (Räsänen et al., 2016).

## Beyond pharmacologic approaches

Nutritional supplementation and exercise training may also exert antioxidant properties (Andreadou et al., 2009; Haykowsky et al., 2009; Scott et al., 2011, 2013; Kirkham and Davis, 2015; Stefani et al., 2015; Singh et al., 2016). While in experimental models, dietary supplementation of antioxidants can mitigate LV dysfunction induced by ANTs (Rephaeli et al., 2007; Andreadou et al., 2009; Xi et al., 2012), evidence suggesting that antioxidant supplementation may modulate ANT-induced CTX in cancer patients is still scant (Fuchs-Tarlovsky, 2013).

Exercise has a positive impact on CV risk factors (e.g., hypertension, high cholesterol and lipids, overweight and diabetes; Kirkham and Davis, 2015) and it has been hypothesized that aerobic exercise can reduce ROS production and restore calcium cycling, thus improving myocardial energetics (Scott et al., 2011). There is some evidence that physical exercise can be beneficial to cancer patients (Stefani et al., 2015). Preliminary studies showed a role for aerobic exercise in combating ANT- (Schermuly et al., 2005) and trastuzumab-induced CTX (Haykowsky et al., 2009). Further studies will be necessary to assess the effects of exercise on CTX caused by anticancer agents (Scott et al., 2013).

## Redox-related biomarkers of cardiotoxicity

One of the main obstacles that renders difficult the prevention of several types of CTX is their complex pathogenesis and lack of reliable biomarkers. Biomarkers ideally should be simple to measure, widely available, low-cost, and used in other pathological conditions. Rather than using single biomarkers, the complexity of CTX is likely to be captured by the association of two or more biomarkers or by modern high-throughout “omics” platform (Chen et al., 2012). At the moment, troponins (Oztop et al., 2004; Suter and Ewer, 2013; Zamorano et al., 2016), brain natriuretic peptide (BNP) and its N-terminal fragment (NT-proBNP), mainly released from cardiomyocytes may be used as biomarkers of CTX in clinical practice (Cardinale et al., 2015; Novo et al., 2016b).

In the setting of cardiac toxicity induced by redox alterations from anticancer drugs, most ROS/RNS are very unstable, with half-lives of 10^−6^–10^−9^s. Also more long-lasting ROS, such as H_2_O_2_, have a half-life of less than a millisecond (Garcia-Garcia et al., 2012). Hence, it is still difficult to assess ROS/RNS generation due to limitations that affect their detection. Therefore, there is a need to identify alternative biomarkers of oxidative/nitrosative CTX. The metabolomic identification of acetate and succinate can be used as a redox-biomarker (Andreadou et al., 2009). Decrease in NAD(P)H:quinone oxidoreductase 1 activity and increased ROS production by NAD(P)H oxidases have been proposed as early biomarkers of LV dysfunction due to ANTs (Novo et al., 2016b). An increase of IL-6 and its soluble receptor (sIL-6R), has been correlated with an early alteration in systolic function in patients treated with EPI (Dessí et al., 2011, 2013). Other potential redox-related biomarkers are high-sensitivity C-reactive protein (CRP), heart-type fatty acid-binding protein (H-FABP), and glycogen phosphorylase BB (GPBB), while some miRNAs that could be used in the assessment of acute coronary syndromes (Novo et al., 2016b) may also be helpful in early detection of CTX (Horacek et al., 2010; Horie et al., 2010; Wang et al., 2013).

## Conclusions and perspectives

Novel anticancer drugs (e.g., targeted therapies and immune checkpoint inhibitors) have revolutioned the management of a wide spectrum of malignancies (Johnson et al., 2016; Menzies et al., 2017; Varricchi et al., 2017b). However, CTX caused by both conventional and novel antineoplastic drugs remains a critical issue (Tocchetti et al., 2013; Ghigo et al., 2016). Chemotherapeutics such as doxorubicin are the prototype of drugs causing CTX (Ghigo et al., 2016). Targeted therapies, initially thought to be safer, can also be responsible of some degree of CTX. Moreover, there is increasing evidence that immune checkpoint inhibitors (i.e., mAbs blocking CTLA-4, PD-1, and PD-L1 on immune cells) can also produce a spectrum of immune-related adverse events, including CTX (Varricchi et al., 2017c,d). Importantly, certain drugs used to prevent cardiovascular complications can even contribute to cancer induction (De Caterina, 2015). Several strategies have been proposed to prevent CTX from antineoplastic agents. None of these is completely safe and satisfactory. This is, at least in part, due to the complexity of different types of CTX. Moreover, it is important to note that heart dysfunction can also manifest years after cancer therapy, making it difficult to evaluate preventive and treatment strategies. It is important to understand the biochemical and molecular mechanisms by which anticancer agents affect cardiomyocytes and immune cells for implementing optimal drug design.

Although oxidative and nitrosative stress elicited by chemotherapeutic agents can harm the heart, indiscriminate elimination of ROS and RNS by antioxidant drugs may not provide beneficial effect, and may even impair physiological cellular functions (Aon et al., 2010; Cortassa et al., 2014; Nickel et al., 2014; Münzel et al., 2015; Arcaro et al., 2016). Indeed, anti-oxidants have been shown to fight LV remodeling and ameliorate contractility in many HF experimental models. Nevertheless, when translated to the clinical arena, these therapeutic approaches did not lead to much benefit or even worsened mortality (Kirk and Paolocci, 2014; Arcaro et al., 2016), when the antioxidant effect was not coupled to other pharmaceutical and biological properties (Fonarow, 2009). Importantly, the site of generation of ROS can determine their biological effects on cardiomyocytes. Hence, more specific, targeted, and “compartmentalized” antioxidant strategies that blunt local ROS/RNS production might be more successful than broad indiscriminate approaches.

In conclusion, although in the last decade research implicating ROS/RNS in antineoplastic drug-induced CTX has greatly advanced, experimental studies and clinical trials are needed to close several gaps in our knowledge of molecular and clinical aspects of CTX in order to balance safety and efficacy of cancer therapy.

## Author contributions

All authors listed have made a substantial, direct and intellectual contribution to the work, and approved it for publication.

### Conflict of interest statement

CT received speaking fees from Alere. The other authors declare that the research was conducted in the absence of any commercial or financial relationships that could be construed as a potential conflict of interest.
